# Maximum likelihood estimation of perceptual differences in sorting tasks

**DOI:** 10.1371/journal.pone.0349396

**Published:** 2026-05-20

**Authors:** Yulong Liu, Huazhi Li, Yali Xiang, Mengni Zhou, Qingqing Li, Hongtao Yu, Yoshimichi Ejima, Satoshi Takahashi, Jiajia Yang, Bin Bai, Xinian Yi, Jinglong Wu

**Affiliations:** 1 Key Laboratory of Intelligent Monitoring on Navigation Safety, Hunan University of Information Technology, Changsha, China; 2 Liaoning Normal University, Liaoning, China; 3 College of Software, Taiyuan University of Technology, Taiyuan, China; 4 School of Education, Wenzhou University, Wenzhou, China; 5 Cognitive Neuroscience Lab, Graduate School of Interdisciplinary Science and Engineering in Health Systems, Okayama University, Okayama, Japan; University of Padova, ITALY

## Abstract

Psychophysical paradigms are foundational for quantifying perceptual discrimination. Yet, a persistent trade-off between assessment efficiency and accuracy limits their broad applicability. To address this, we introduce a novel evaluation model grounded in maximum likelihood estimation (MLE) for perceptual sorting tasks. This work details the model’s formulation, validates its performance through simulation, and demonstrates its efficacy in a tactile angle-sorting experiment. Our findings reveal that the sorting paradigm, particularly with five stimuli across three trials, achieves an optimal balance of efficiency and robustness. This method provides a potentially useful and relatively efficient approach for assessing perceptual discriminability within the tactile experimental context, with preliminary indications of its applicability in both research and practical screening.

## Introduction

Perceptual discrimination, a cornerstone of human interaction with the environment, has long been quantified using psychophysical methods [1–6]. These methods hold particular promise for clinical screening of cognitive decline. Yet, a critical bottleneck persists: widely adopted paradigms like the two-alternative forced-choice (2AFC) are often too time-consuming for practical, large-scale application, especially among elderly or clinical populations [7–10]. This gap between laboratory precision and clinical feasibility motivates our search for more efficient paradigms [11–16].

Incorporating these assessments into disease diagnosis offers a more comprehensive insight into the interplay between the accuracy of subjective perception of objective stimuli and the health of neurons or nervous systems [10,17,18]. This perspective holds significant value in advancing our understanding of cognitive and neural health. For example, Yang et al. employed a tactile angle discrimination system to assess the ability to perceive tactile angles among patients with mild cognitive impairment (MCI), Alzheimer’s disease (AD), and healthy individuals. Their findings revealed a significant difference in angle discrimination thresholds between AD patients and healthy controls. Moreover, MCI patients also exhibited a notably reduced ability to discern tactile angles compared to healthy individuals. These results establish a reliable benchmark for clinical screening measures [14]. As certain groups, such as the elderly and individuals with AD or HD, often exhibit challenges in enduring prolonged cognitive tasks, it becomes imperative to identify efficient and convenient testing methods for widespread applicability. However, there exists a limited number of assessment methods for perceptual discrimination ability that meet these dual criteria.

The widely adopted experimental paradigms for measuring perceptual discrimination include the comparative approach, such as the two-alternative forced choice (2AFC), three-alternative forced choice (3AFC), or adaptive staircases. These methodologies rely on multiple iterations of pairwise comparisons to derive accuracy metrics, which are subsequently fitted to mathematical models, such as the logistic function or Gaussian cumulative function. The threshold within the model is then computed to assess individual discriminative performance. Despite their efficacy in scientific research or clinical screening, these methods pose challenges for large-scale clinical applications. In order to obtain better discrimination performance, this type of experimental paradigm requires calculating accuracy for multiple trial comparisons, often exceeding ten and extending test times. While valuable, the increased trial demands hinder convenience, presenting a barrier to widespread acceptance of these tests. Hence, there is a pressing need for experimental paradigms that strike a balance between efficiency and accuracy, coupled with the corresponding mathematical models that underpin them. This requirement is crucial for both academic research and the practical application of disease diagnosis.

Building on the challenges above, our study addresses touch perceptual discrimination assessment by proposing a novel sorting task paradigm evaluated via a maximum likelihood estimation (MLE) framework. The choice of a sorting task over established paradigms is motivated by several key considerations. First, it offers superior assessment efficiency. Unlike the 2AFC method, which extracts information from binary comparisons, a single sorting trial with *n* stimuli implicitly contains information from multiple pairwise comparisons simultaneously, offering higher data density per unit time. Our prior work demonstrated that a stable discrimination index can be obtained in approximately 10–15 minutes using sorting, compared to 2.5–3 hours for a classic 2AFC threshold measurement, representing an order-of-magnitude improvement in speed [9]. Second, it provides a stable estimate of perceptual standard deviation (σ) across a stimulus range. While adaptive staircases excel at finding a single threshold point quickly, the sorting paradigm with a fixed set of equally spaced stimuli allows for the direct estimation of the underlying perceptual noise parameter (σ) across multiple levels, which may offer a more robust measure of an individual’s general discriminability in that dimension. Third, it possesses greater ecological validity for complex cognition. Organizing multiple items based on a perceived property is a common real-world cognitive operation, involving not only sensory discrimination but also working memory and executive function to some degree. This makes it potentially more sensitive to higher-order cognitive decline. Lastly, the paradigm’s simplicity makes it highly accessible for non-expert administration in clinical or field settings. The goal of this work is not to replace traditional psychophysical methods, which remain the gold standard for detailed mechanistic studies, but to provide a complementary tool optimized for efficient, stable, and practical assessment of perceptual discrimination ability, particularly in applied and screening contexts.

This study aimed to explore the optimal relationship between haptic perceptual discrimination performance and the number of stimuli and trials, and further validate the efficiency and stability of angle sorting assessment methods. Here, we first present the principle and calculation method of the MLE model. We applied a Gaussian model to the neural pathways underlying angular stimulus feature perception to explain the strength of perceptual responses. And based on Gaussian function, we constructed a maximum likelihood estimation (MLE) assessment model to calculate the standard deviation (σ), which is used to measure individual perceptual discrimination ability in a stimulus sorting paradigm. In this paper, we first present the principle and calculation method of the MLE model. Then, conducted a parameter-recovery simulation experiment about σ. We simulate the subjects to complete the ranking task through computer simulation. Based on the sorting results completed by simulation, we systematically evaluate the evaluation performance of the model, including the influence of the number of experiments and the number of angle stimuli on the stability of the evaluation results for individual and group subjects in the angle sorting paradigm. Subsequently, based on the simulation results, we further verified the stability and effectiveness of the model in testing individual discrimination ability by recruiting subjects to complete the angle sorting experiment.

## Theoretical model

### Normal distribution

Normal distribution probabilistic methods have often been applied in modern psychophysical research [19]. The most famous for its use in Thurstonian modeling is the use of a normal distribution [20–23]. The theory model has widely applied in the field of psychology, such as in the assessment of sensory discrimination [22–27]. The premise on which the model is established typically assumes that individuals’ discriminative abilities towards a fixed set of equally spaced discrete observational samples are stable. Individuals evaluate each observed sample through perception, and the actual observed result is compared or contrasted with its intrinsic psychological-physical quantity, thus facilitating recognition of the observed samples. Here, the psychophysical quantities compared are the correct observation samples. Based on the above assumptions, a normal distribution model is constructed, in which the stable discrimination ability is the standard deviation (σ) of the model; the equidistant scattered observation samples identified by the individual are the independent variables (*x*) corresponding to the model; and the compared psychophysical quantities (the correct observation samples in comparison) represent the mean (μ) of the model; the final calculation result (y=f(x,u,σ)) of the normal distribution function represents the reaction intensity of different observation samples when $x_{\text{0}}=\mu$ is evaluated [22,28]. The normal model function is as follows:


$$y=f\left(x,u,\sigma \right)=\frac{\text{1}}{\sigma \sqrt{2\pi }}e^{-\frac{\text{1}}{\text{2}}{\left(\frac{x-\mu }{\sigma }\right)}^{\text{2}}}$$ (1)

In formula (1), the σ represents the standard deviation which we call the discrimination index (DI) used to assessment one individual’s perceptual discrimination ability. Guided by the Thurstonian framework of comparative judgment, we model the internal representation of each touch angle as a random variable following a normal distribution. Conceptually, this captures the trial-to-trial noise inherent in sensory processing. The spread of this distribution, quantified by its standard deviation σ, is central to our model. σ is a dimensionless quantity that represents the standard deviation (σ) resulting from the difference between the sequence of sorting tasks completed by an individual subject and the correct sequence. In the model, the comparative psychophysical quantities μof the observation samples always vary with the sample of observations varying among equidistant scatters. And since the standard deviation is constant, this normal model is then mathematically just a horizontal translation over the values of the observation samples. Thus, we find that the panning process always corresponds to a peak at $x_{\text{0}}=\mu$, i.e., the response intensity is highest at the observation sample. We define this phenomenon as the observation sample $x_{\text{0}}$ ($x_{\text{0}}=\mu$) in the perceptual channel CH $X_{\text{0}}$. In the channel CH $X_{\text{0}}$, the response intensity to the observation sample $x_{\text{0}}=\mu$ is the largest, while the response intensity of the other observation samples decreases (both left and right) as the values of the observation samples obey a normal distribution function.

**Fig 1:**
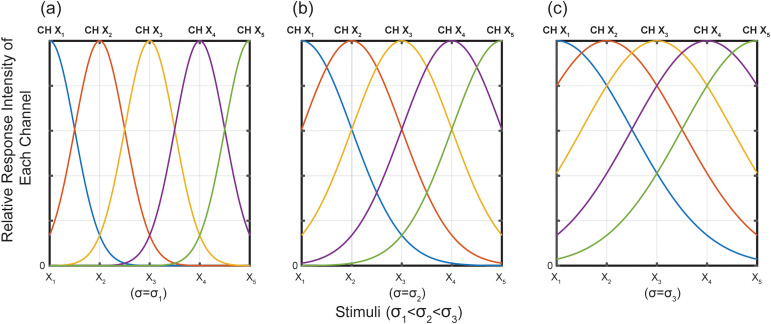
Each channel of the participants corresponds to the relative response intensity of the observations for different CH values. For the same stimulus at the same CH, the relative response intensity differed among participants. For example, in CH $X_{\text{1}}$, three participants with $\sigma =\sigma_{\text{1}}$ (a), $\sigma =\sigma_{\text{2}}$ (b) and $\sigma =\sigma_{\text{3}}$ (c) responded differently to stimulation $x_{\text{2}}$ (the orange line). The relative response intensity is maximum only when the stimuli $x_{i}$ and CH $X_{i}$ match.

In the assessment of perceptual discrimination, we employed a normal distribution model to elucidate the connection between the observed samples and the model parameters. However, perceptual discrimination not only relies on such mathematical models, but is also closely related to cognitive psychology. In the process of cognitive perception of the external environment, the human brain initially receives information through one or more sets of sensory receptors and then transmits this information to the brain via one or more ascending spinal cord neurons. Following the processing of information in the primary and secondary sensory areas as well as in higher cognitive regions, the brain ultimately forms its understanding. Consequently, cognitive processes are contingent upon the transmission of information through one or more sensory neural pathways [29]. These pathways encompass sets of sensory receptors, spinal cord neurons responsible for relaying information upwards, and specific brain regions involved in receiving and processing sensory input. The qualitative and quantitative dimensions of perceived experience are determined by the relative activity levels within these pathways [30]. To further quantify the observed samples, we propose a hypothesis regarding the quantified physical properties of the observed samples. It is assumed that each observed sample $\left(x_{i}\right)$ in the samples sequence has a unique combination of neural recognizing mechanisms which we name Channel $X_{i}$ (CH $X_{i}$); the participant use CH $X_{i}$ to estimate each actual physical quantities. When each participant with a different standard deviation (σ) evaluates the same sequence series of observed samples, the relative intensity of responses to the same set will also be different, as shown in Fig 1 [22].

**Fig 2:**
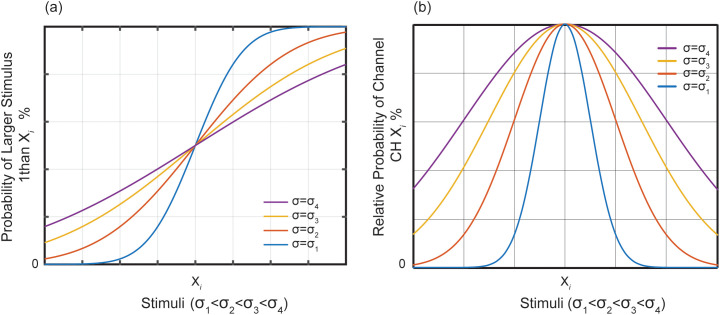
The relationship between different stimuli$x_{i}$ and the neural pathways CH $X_{i}$ corresponding to them. (a) The probability that the participants correctly determined the characteristics of $\begin{array}{c} x_{i} \end{array}$ had a cumulative normal distribution that corresponded to CH $\begin{array}{c} X_{i} \end{array}$. (b) The relative probability of CH $\begin{array}{c} X_{i} \end{array}$ for different participants in response to different stimuli.

At the same time, the percentage of relative response intensity activated by the corresponding physical observed sample($x_{i}$) through CH $X_{i}$ has a cumulative normal distribution corresponding to CH $X_{i}$, as shown in Fig 2(a). The normal cumulative function varies slightly from the logistic function model of the continuous stimulus method for measuring thresholds. Although the set of levels independent of the distinct parameters does not influence the outcomes of the assessment analyses due to these two types of methods, their cumulative distributions form statistical predictions under the method of constant stimuli [31].

Therefore, the individual differences of discrimination ability among participants are represented through the standard deviation (σ) or DI of a normal distribution by the relative response intensity of the different CH $X_{i}$, as shown in Fig 2(b); it is also possible to predict the discrimination threshold under the method of constant stimuli methods using the same normal cumulative distribution, as shown in Fig 2(a). Participants compare the characteristics of observed sample one by one and then sort them according to the rules in the sorting task. To facilitate the analysis of results, the psychological experimental result is assumed step by step using a unilateral starting method, upper-or lower-sided the observed samples, without paying attention to the actual process of completing the sorting task. In fact, no matter where you start to analyses, it will not influence the analysis results.

### Maximum Likelihood Estimation (MLE)

#### Foundational assumptions of the model.

The model is built upon four core assumptions, extending principles from Thurstonian comparative judgment law and signal detection theory to the sorting task paradigm [20–23,32].

**Assumption 1: Gaussian Perception Model.** The psychological perception of an individual towards a single physical stimulus follows a normal distribution. The psychological perception of an individual to a single physical stimulus follows a normal distribution. For a stimulus with a physical value of $x_{i}$, the distribution of its internal perceptual representation $\psi_{i}$ is:


$$\psi_{i}\sim \;N\left(\mu_{i},\sigma^{\text{2}}\right).$$ (2)

In formula (2), $\mu_{i}$ corresponds to the physical value $x_{i}$ from CH $X_{i}$, and σ represents the standard deviation of the perception process. This σ is the core parameter to be estimated, reflecting the inherent variability or “noise” in perception.

**Assumption 2: Perceptual Channel Independence.** In the single-comparison judgment of a sorting task, an individual’s perception of the currently evaluated stimulus is independent of their perception of other stimuli. For a series of stimuli $𝐒=\left\{S_{\text{1}},S_{\text{2}},...,S_{k}\right\}$ arranged equidistantly in physical dimensions, an individual possesses k corresponding “perceptual channels.” When attempting to identify the current stimulus as $S_{i}$, all channels are activated, with the response intensity represented by the probability density function value of the stimulus in that channel. And the individual is assumed to select the $S_{i}$ stimulus corresponding to the channel with the highest current response intensity as their perceptual judgment. At this point, the distribution of response intensities across channels for stimulus $S_{i}$ forms a normal probability density function centered at u and scaled by σ. This assumption allows each independent perceptual evaluation of different stimuli to be statistically independent. That is, the random fluctuations in internal perceptual representations $\psi_{i}$ and $\psi_{j}$ (i≠j) are statistically independent, ensuring the model can decompose the joint probability of multiple comparisons into the product of independent probabilities.

**Assumption 3: Sequential Decision-Making.** To facilitate modeling, we simplify the complex dynamic ordering process into a sequential decision-making process. Starting from the leftmost (smallest) position, we sequentially select the perceptually “smallest” stimulus from the remaining unsorted stimuli to fill each vacancy. This assumption decomposes the generative probability of a sequence into a product of conditional probabilities. This does not compromise the statistical consistency of model estimates, as any final ordering outcome can be mapped to a specific sequential decision path.

**Assumption 4: Perceptual Stability in Individuals.** During the completion of a sorting task involving a specific set of stimuli, an individual’s perceptual level σ remains constant and does not undergo systematic changes due to fatigue, learning, or the position of stimuli within the sequence.

#### Modeling perceptual signals and sequential decisions.

Consider a set of *n* stimuli with physically equal increments, ordered as $𝐒=\left\{S_{\text{1}},S_{\text{2}},...,S_{N}\right\}$, where $S_{\text{1}}<S_{\text{2}}<...<S_{N}$.The goal for the participant is to arrange these stimuli in ascending order. The observed sorting result (Actual Sort, 𝐀𝐒) is a sequence $𝐀𝐒=\left[a_{\text{1}},a_{\text{2}},a_{\text{3}},\cdots ,a_{n}\right]$，where $a_{k}$ is the stimulus identifier placed in the *k*th position.

Based on Assumptions 1 and 3, when making a decision for position the kth position, the participant faces the remaining unsorted stimulus set $R_{k}$. He must select the stimulus perceived as the smallest from this set. For any candidate stimulus $S_{j}\in R_{k}$ the probability of it being perceived as “currently smallest” depends on the joint probability that its perceived magnitude $\psi_{j}$ is smaller than the perceived magnitudes $\psi_{i}$(∀i≠j) of all other stimuli in the set $R_{k}$. To simplify calculations, we introduced an equivalent decision rule based on an “ideal observer”: participants estimate the probability that each remaining stimulus $S_{j}$ is the “current correct choice” (i.e., the stimulus $S_{min\left(R_{k}\right)}$ with the smallest physical quantity in set $R_{k}$). According to Assumption 1, the strength of this probability can be measured by the value of the probability density function of the perceived quantity $\psi_{j}$ at $\mu_{min}$ (corresponding to the psychophysical quantity $S_{min\left(R_{k}\right)}$). Therefore, the relative likelihood of stimulus $S_{j}$ being selected at step k is proportional to the probability density value of its perceived quantity distribution:


$$P(S_{j}|R_{k},\sigma )\propto f(S_{min\left(R_{k}\right)};\mu =S_{j},\sigma ).$$ (3)

In formula (3), f(⬝) is the probability density function of a normal distribution.

#### Constructing the likelihood function.

Given the result 𝐀𝐒 of a sorting task and the model parameter $\hat{\sigma }$ to be estimated, we can calculate the (conditional) probability of that specific sequence occurring. At step kth, the remaining stimulus set is $R_{k}$, and the stimulus actually selected is $a_{k}$. The probability $P_{k}$ is obtained by normalizing the density value for $a_{k}$ against the sum of density values for all stimuli in $R_{k}$.

For instance, at position CH $X_{\text{1}}$ (choosing the first element $a_{\text{1}}$): The remaining set $R_{\text{1}}$ is the full set of stimuli. The probability of selecting $a_{\text{1}}$ is:


$$P_{\text{1}}\left(\sigma ,a_{\text{1}}\right)=\frac{f\left(a_{\text{1}},\mu =S_{\text{1}},\sigma \right)}{\overset{j=n}{\underset{j=1}{\sum }}f\left(S_{j},\mu =S_{\text{1}},\sigma \right)}.$$ (4)

In formula (4), $S_{\text{1}}$ is the physically smallest stimulus (the correct choice for the first position). This logic extends to calculating the probability $P_{k}\left(\sigma ,a_{k}\right)$ for any stimulus $a_{k}$ being the first choice.

The posterior probability that the observed choice $a_{\text{1}}$ is selected, given the model and parameter σ, is the ratio of its likelihood to the sum of likelihoods for all stimuli at that position:


$$P_{a_{\text{1}}}=\frac{P_{\text{1}}\left(\sigma ,a_{\text{1}}\right)}{\overset{j=n}{\underset{j=1}{\sum }}P_{\text{1}}\left(\sigma ,a_{j}\right)}.$$ (5)

In formula (5), there is Normalization of Probabilities at Position CH $X_{\text{1}}$.

At subsequent positions (e.g., position CH $X_{\text{2}}$), once a stimulus has been selected, it is removed from the remaining set. Therefore, for position $X_{\text{2}}$, the remaining set is $R_{\text{2}}=𝐀𝐒\;\left\{a_{\text{1}}\right\}$. The probability of selecting a stimulus $a_{\text{2}}$ is calculated based on the new smallest stimulus in $R_{\text{2}}$, which is $S_{min\left(R_{\text{2}}\right)}$ (likely $S_{\text{2}}$ if $a_{\text{1}}$ was correct, $a_{\text{1}}=S_{\text{1}}$, or another value if not). Crucially, any stimulus that has already been placed (like $a_{\text{1}}$) has a probability of 0 of being selected again at position CH $X_{\text{2}}$. For example, the probability of selecting $a_{\text{2}}$ at position CH $X_{\text{2}}$ is:


$$P_{\text{2}}\left(\sigma ,a_{\text{2}}\right)=\frac{f\left(a_{\text{2}},\mu =S_{min\left(R_{\text{2}}\right)},\sigma \right)}{\sum_{S_{j}\in R_{\text{2}}}f\left(S_{j},\mu =S_{min\left(R_{\text{2}}\right)},\sigma \right)}.$$ (6)

In formula (6), the denominator sums over all stimuli in $R_{\text{2}}$. The posterior probability for the observed choice $a_{\text{1}}$ (which could be $S_{\text{2}}$, $S_{\text{3}}$, etc.) is calculated similarly.

The posterior probability that the observed choice $a_{\text{2}}$ is selected, given the model and parameter σ, is the ratio of its likelihood to the sum of likelihoods for all stimuli at that position:


$$P_{a_{\text{2}}}=\frac{P_{\text{1}}\left(\sigma ,a_{\text{2}}\right)}{\overset{j=n}{\underset{j=2}{\sum }}P_{\text{2}}\left(\sigma ,a_{j}\right)}.$$ (7)

In formula (7), there is Normalization of Probabilities at Position CH $X_{\text{2}}$.

In general, for the *k*th position CH $X_{k}$(k≠n), the remaining set $R_{k}$ consists of all stimuli not chosen in the previous (*k* − 1) positions. The probability of selecting stimulus $a_{k}$ (which is the actual observation) is the formula (8), as follows:


$$P_{k}\left(\sigma ,a_{k}\right)=\frac{f\left(a_{k},\mu =S_{min\left(R_{k}\right)},\sigma \right)}{\sum_{S_{j}\in R_{k}}f\left(S_{j},\mu =S_{min\left(R_{k}\right)},\sigma \right)}.$$ (8)

The observed choice $a_{k}$ is selected, given the model and parameter σ, is the ratio of its likelihood to the sum of likelihoods for all stimuli at that position:


$$P_{a_{k}}=\frac{P_{k}\left(\sigma ,a_{k}\right)}{\overset{j=n}{\underset{j=k}{\sum }}P_{k}\left(\sigma ,a_{j}\right)}.$$ (9)

In formula (9), there is Normalization of Probabilities at Position CH $X_{k}$.

At the *n*th position CH $X_{n}$, the remaining set $R_{\text{1}}$ contains only a single stimulus $a_{n}$. At this point, the probability is 1, that is $P_{a_{n}}=1$.

Based on assuming the sequential decisions are conditionally independent (based on Assumption 2), the likelihood of observing the entire sequence AS, given the parameter σ, is the product of the posterior probabilities for the choice made at each position. So, the likelihood function for the full sequence is:


$$ℒ\left(\sigma |𝐀𝐒\right)=P\left(𝐀𝐒|\sigma \right)=\prod_{k=1}^{n}P_{a_{k}}.$$ (10)

In formula (10), $P_{a_{k}}$ is the normalized posterior probability of the observed choice $a_{k}$ at step *k*.

#### The evaluation parameter.

Based on the constructed likelihood function, we compute the maximum value of the estimated individual discrimination index ${\hat{\sigma }}_{MLE}$:


$${\hat{\sigma }}_{MLE}=\underset{\sigma >0}{argmax}ℒ\left(\sigma |𝐀𝐒\right).$$ (11)

In the context of this psychophysical assessment, this estimated ${\hat{\sigma }}_{MLE}$ is defined as the Discrimination Index (DI) for the participant in formula (11). A larger DI indicates greater variability in perception (a larger ${\hat{\sigma }}_{MLE}$), meaning the participant’s sorting results deviate more from the correct order, signifying poorer discrimination ability. Conversely, a smaller DI indicates more precise perception and better discrimination ability, as the participant’s internal representations are more tightly clustered around the true stimulus values. A perfectly correct sort would yield the smallest possible DI for that stimulus set.

Among these, the estimated ${\hat{\sigma }}_{MLE}$ represents the standard deviation of individual perception and also serves as the core parameter DI in the estimation. It shares a theoretical connection with the difference threshold (DT) in classical psychophysics. In a normal perception model with μ as the reference, the DT corresponding to a 75% accuracy rate is $DT_{transfer}=z_{0.75}\cdot \sigma \approx 0.675\sigma$. Therefore, DI is a measure directly proportional to the JND, where smaller values indicate sharper perceptual discrimination ability. This demonstrates that DI is not merely a statistical parameter of the model but also an effective estimator of an individual’s fundamental perceptual discrimination capability. In our prior research, we experimentally confirmed that the threshold angle $DT_{transfer}$ derived from the DI estimated via the ranking task exhibits a high correlation (r=0.9075) with the angle DT measured by the traditional two-alternative forced choice (2AFC) method, providing empirical support for its validity [9].

#### Typical outcomes of the likelihood function.

Depending on the observed sorting sequence 𝐀𝐒, the likelihood function ℒ(σ|𝐀𝐒)(Equation 10) exhibits one of three characteristic patterns as a function of σ.

**Fig 3:**
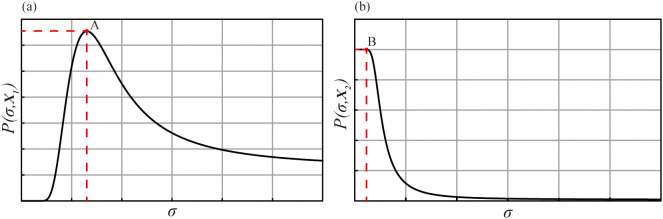
The MLE function linking the probabilityP(σ,𝐀𝐒) and σ for Type I and Type II results. The horizontal axis in the figure represents $\begin{array}{c} \sigma \end{array}$, and the vertical axis represents the probability that corresponds to different $\begin{array}{c} \sigma \end{array}$ values when sorting.

Type I results occur when the sorting result contains tolerable errors (e.g., $𝐀𝐒=\left[S_{\text{1}},S_{\text{3}},S_{\text{2}},S_{\text{4}}\cdots S_{n}\right]$). The likelihood function ℒ(σ|𝐀𝐒) initially increases with σ, reaches a unique maximum, and then decreases. The σ at this peak is the MLE, providing a clear DI value to evaluate the participant’s performance, it is represented by point A in Fig 3(a).

Type II results occur when the sorting observation result is completely correct (e.g.,$𝐀𝐒=\left[S_{\text{1}},S_{\text{2}},S_{\text{3}},S_{\text{4}}\cdots S_{n}\right]$). In this type of result, the probability ℒ(σ|𝐀𝐒) is stable at 1 as σ $\left(0,\sigma_{crit}\right]$ increases within a specific range. And once when σ reaches a critical point $\left(\sigma_{crit},+\infty \right)$, the probability begins to gradually decrease. We adopt this critical value $\sigma_{crit}$ as the final evaluation result DI. This value is used to assess the participant’s performance and is represented by point B in Fig 3(b).

**Fig 4:**
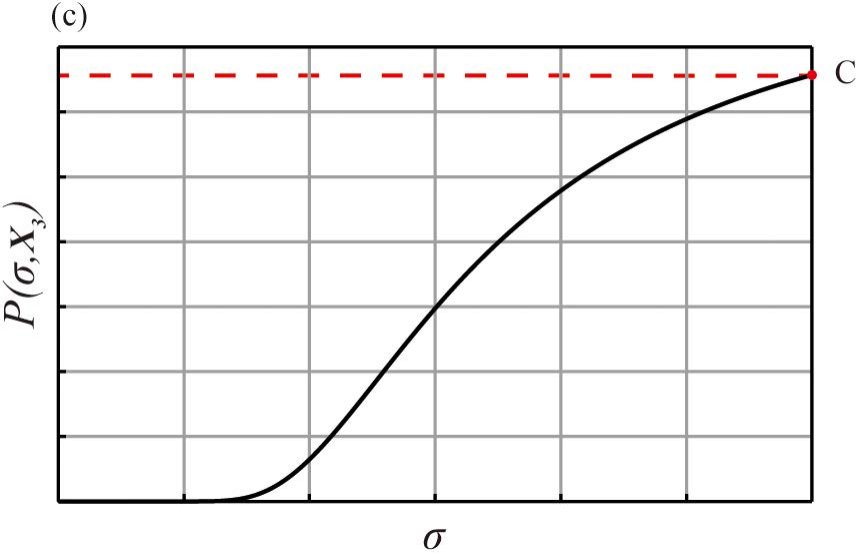
The MLE function linking the probabilityP(σ,𝐀𝐒) and σ for Type III results. The horizontal axis in the figure represents $\begin{array}{c} \sigma \end{array}$, and the vertical axis represents the probability that corresponds to different $\begin{array}{c} \sigma \end{array}$ values when sorting $\begin{array}{c} \text{AS} \end{array}$.

However, the third type of result, termed Type III results, cannot yield a reasonable σ using this model. This occurs when the sorting result is highly inconsistent with the correct order, such as a complete reversal $𝐀𝐒=\left[S_{n},S_{n-1},S_{n-2},\cdots S_{\text{1}}\right]$. In this scenario, the likelihood function ℒ(σ|𝐀𝐒) increases monotonically with σ and does not reach a maximum or a plateau, as shown in Fig 4. The model fails to estimate a finite DI. This outcome suggests that the task was too difficult for the participant, possibly because the perceptual differences between stimuli were too small. The recommended course of action is to repeat the task with stimuli that have larger physical differences.

According to this model, we used MATLAB (MATLAB Inc.) to simulate all probabilities within the control σ range and to diagram their relationships. The MATLAB code used to determine the parameters of this model has been uploaded to Open Science Framework [33].

## Computer simulation

### Data preparation

To obtain the real DI data of the subjects in the simulation experiment, we randomly generated the real DI of the healthy population using the gaussian function. Here, we constructed a simulated model that considers only numerical values as stimuli, which is based on simulated participants’ performance in discriminative ability in a sorting task, in which participants have the following characteristics:

1. Constant DI: TheDI of the participant is constant and does not change due to changes in the number of observations;
2. True DI Calculation: Considering the significance of the practical application of DI, all subjects assumed that the real DI is greater than 0; in addition, according to the results of previous research assessments, the discrimination using an angle of 2° is more appropriate [9,34]. In the setting of the simulation, the σ range is completely sorted correctly when the evaluation of the σ is the minimum value, the positive value of which is subject to a normal distribution. The true σ of the participant group, simulated by the computer, is given by the formula (12), as follows:

σ=|a|+x (12)

where a~N(1,1) is drawn from a normal distribution with a mean of 1 and a standard deviation of 1, and x is a constant value that applies when the sorting is performed completely correctly. This setting is an approximation we fitted based on previous research, using the correlation between the angle discrimination threshold measured by 2AFC and the equivalent angle threshold of the DI estimated by sorting. The goal is to generate a DI with a reasonable distribution of healthy people [9].

3. Simulation Process: The computer simulation of a participant completing the sorting task follows the model described in this paper (The Section-The Evaluation Parameter σ). After determining the real DI for the participant population, the sorting process is simulated using the previously calculation σ. During the sorting process, observation samples are placed from left to right, and the probability of placing each sample in its respective position is calculated based on the real DI. The observation values are then sorted according to these probabilities;
4. The minimum value of the true σ is cut off, however, the results of individuals repeating the test evaluation σ many times must obey the normal distribution, which will lead to a great reduction of the σ. To avoid this result, in the simulation, we strictly control the evaluation σ obtained when the ordering is completely correct—i.e., in the simulation experiments, we required ten consecutive perfect correct before obtaining the final evaluation DI of the participants in type II;
5. In addition, when a simulated subject’s sorting result appeared to be type III, the angular stimulus would be increased by 1.5 times the interval value before completing the sorting until a sorting trial ended when the sorting assessment resulted in a type I or type II result (ten consecutive if correct sorts) [9,14,15,34–36].
6. Last but not least, simplifying theoretical assumptions through computer models by adding constraints often makes it difficult to fully reproduce the complexity of human behavior. For example, ignoring individual differences or non-linear psychological processes. These performances may underestimate the diversity of behavior, simplifying the decision-making of simulation models to purely rational calculations. This may lead to model simplification errors in computer simulations of human behavior. Therefore, a correction coefficient λ is introduced in the simulation process of the sorting task.

### Data processing and analysis

We used the evaluation algorithm in MATLAB (MathWorks Inc.), described in Section-The Evaluation Parameter σ, to compute the results of the computer simulated sorting task and obtained the DI for each sorting trial. The number of simulated trials that were completely correct (Type II results) and Type III results out of all simulated trials is shown in Table 1.

**Table 1:** The number of correct (Type II) and invalid (Type III) trials in the assessment results simulated for all participants in the first sorting.Number of stimuli.

	3	4	5	6	7	8	9	10
Type II/trials	75740	73944	73551	73231	72886	72994	72906	72964
Type III/trials	4765	1038	179	18	0	0	0	0

Next, we excluded the Type III results and evaluated the average DI of the valid results after each trial was completed. Here, to assess the impact of the number of trials on the results, we calculated the nth average biased and corrected DI using the formula (13), as follows:


$${\text{DI}}_{nm}=\frac{\overset{i=n}{\underset{i=1}{\sum }}{\text{DI}}_{i}}{n},\left(1\leq n\leq 10\right)$$ (13)

for each simulated participant under each condition; the $DI_{i}$ is the evaluation value calculated by substituting the sorted data completed for the *i*-th time into the evaluation model. We then calculated the error and percent error between the average DI after each sorting task and the true DI using the formula (14), as follows:


$$Percentage\;Error=\frac{Error}{DI_{t}}=\frac{\left|\lambda DI_{mn}-DI_{t}\right|}{DI_{t}}$$ (14)

where $DI_{t}$ is the true value, and $DI_{mn}$ is the average evaluation value sorted *n* times. We calculated the average percentage error for different numbers of trials in each case. Additionally, Furthermore, in the simulation experiment, based on empirical rules, the individual evaluation deviation is assumed to be the standard normal deviation. To obtain a more accurate evaluation DI value, the correction coefficient λ is determined based on the distribution of the ***Z***-score, that is, the deviation is controlled by the value $\varepsilon =\Phi \left(\frac{\text{1}}{\lambda }\right)-\Phi \left(-\frac{\text{1}}{\lambda }\right)$. Here, we select four deviation ranges and calculate their corresponding correction coefficients$\frac{\text{1}}{\lambda }=$1.15, 1.28, 1.44 or 1.65, corresponding to ε=0.75, 0.80, 0.85 or 0.90 respectively. According to Formulas (14) and (15), we calculate the percentage errors corresponding to different conditions.

### Results

In this section, we examine the relationship between the accuracy of the model and various experimental parameters, including the number of stimuli, the number of trials, the correction coefficient, and the evaluation percentage error. We investigate how these factors influence model performance and optimize these parameters of the model by comparing the differences in error data across different conditions.

***Efficiency.*** To examine the power to distinguish eight conditions—where participants complete the sorting task with three, four, or five angles—we analyzed the variation in the number of valid results based on the number of stimuli used in a full permutation. Mathematically, the total number of possible outcomes for these conditions is $\overset{\text{3}}{\underset{\text{3}}{A}}=6$, $\overset{\text{4}}{\underset{\text{4}}{A}}=24$, $\overset{\text{5}}{\underset{\text{5}}{A}}=120$, $\overset{\text{6}}{\underset{\text{6}}{A}}=720$, $\overset{\text{7}}{\underset{\text{7}}{A}}=5,040$,$\overset{\text{8}}{\underset{\text{8}}{A}}=40,320$, $\overset{\text{9}}{\underset{\text{9}}{A}}=362,880$, and $\overset{10}{\underset{10}{A}}=3,628,800$. The number of valid (Type I and Type II) Dis calculated by the evaluation model were 3 (50%), 12 (50%), 63 (52.5%), 421 (58.47%), 4,051 (60.54%), 25,165 (62.41%), 297,344 (81.94%) and 2,381,612 (65.63%) for the 3–10 angles conditions, respectively. Although the proportion of type III is relatively high under all conditions, the following methods can obtain valid evaluation results. To obtain effective test results, we cloud increase the diversity of test results by adding multiple sets of stimuli with different spans, making the type of evaluation results more continuous, and reducing the invalid results of a single test (type III). As described in the simulation experiment conditions. However, this may seriously affect the efficiency of the test when real participants complete the experiment. Therefore, it is expected that effective evaluation results can be obtained quickly and accurately with as few sorting times as possible.

***Number.*** Although the increasing in the number of stimuli used makes the evaluation results tend to be continuous, it will also increase the number of participants’ necessary judgments and comparisons, resulting in the extension of the test time. It requires a careful balance between testing efficiency and result accuracy. While more stimuli could enhance the model’s discrimination power and reduce error, it may also introduce participant fatigue and increase cognitive load, potentially impacting test reliability. Therefore, optimizing the number of stimuli is essential to ensure that testing remains both efficient and effective in producing accurate assessment results.

***Spacing and Trials.*** Increasing the stimulus interval could also obtain valid assessment results, but the method of multiple selection of stimulus intervals and multiple trial counts would have no testing advantage over other testing methods in practice, and would also pose other problems. On the one hand, the perceived adaptation of a smaller number of trials has a smaller impact on the results of the experiment, and once the stimulus interval is increased leading to differences in perceived adaptation does not facilitate the comparison of assessment results. In addition, obtaining a suitable interval through multiple trials ensures that the assessment results are valid which greatly reduces the efficiency of the test compared to a single trial which yields valid results.

**Fig 5:**
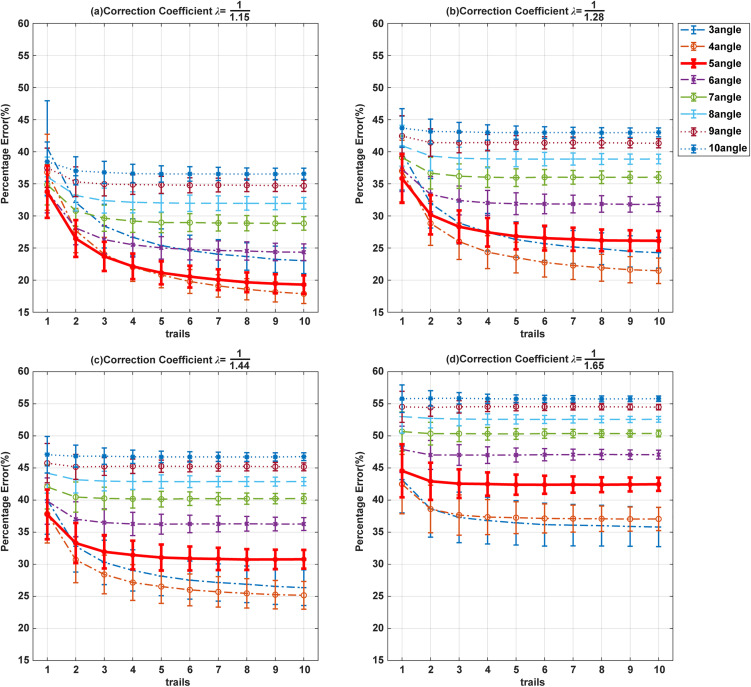
Percentage error when sorting varying numbers of stimuli in computer simulation. Figures (a)~(d) represent the percentage error after 1 ~ 10 trials for different the correction coefficient λ.

***Percentage Error.*** To study the error in the measurement model, we calculated all simulations of the valid results for estimator DI across all participants in eight conditions, along with the corresponding percentage error. We then generated a displaying the percentage error observed after each sorting task under various conditions, as shown in Fig 5.

### Discussion

Through simulation studies, we investigated the applicability of the MLE estimating model when assuming large-scale data simulation. The model’s evaluation performance was examined after each trial under eight conditions to assess its power and percentage error. Although the proportion of effective results in the evaluation utility of all theoretical conditions is about half, the conditions are set according to the simulation sorting task. When the participants cannot obtain the effective evaluation σ in simulation sorting, that is the σ is type III, they will increase the stimulation interval to complete the sorting again until the effective evaluation σ is obtained. Increasing the number of angular stimuli in the sorting task introduces a greater variety and continuity of σ results, which improves the grading performance of evaluation model. At the same time, it also effectively reduces the adjustment of stimulus interval due to type III in the sorting result and completes the sorting task again. However, simulations may often differ from actual applications. In fact, participants complete the sorting task by making multiple comparisons, when increasing the number of stimuli will not only prolong the test time and reduce the test efficiency but may also cause perceptual adaptability and affect the evaluation results.

As predicted, the simulation revealed that the percentage error in estimating DI monotonically decreased with an increasing number of trials, as shown in Fig 5. Notably, the most substantial gain in precision occurred within the first three trials, suggesting diminishing returns beyond this point. This finding directly informs our experimental design, leading us to prioritize efficiency by limiting the number of trials in the subsequent behavioral study.

## Angle sorting experiment

In this experiment study, we aimed to further explore the relationship between the number of stimuli and the number of trials to determine how these factors influence the robustness of sorting results in practice. We asked participants to complete a sorting task using different numbers of raised-angle stimuli [9]. During the task, participants arranged a set of raised-angle blocks (38×38mm) [34] from left to right, in order from the smallest angle to largest angle. The perception of these stimuli followed the Weber-Fechner law. The DI was calculated using the proposed model to assess touch discrimination performance. We compared performance differences in the sorting tasks across the three block conditions to evaluate how the number of stimuli affected sorting accuracy.

### Participants and methods

#### Ethics statement.

The study was conducted in accordance with the principles of the Declaration of Helsinki and was approved by the Institutional Review Board at Okayama University (Protocol Number: Kan1702−030, date of first approval: 26 May 2020, and date of recent approval: 27 April 2021). Written informed consent was obtained from all participants prior to their involvement in the study. None of the participants had prior experience with the haptic angle sorting test.

#### Participants.

Eighteen healthy right-handed participants (8 males and 10 females, age range: 22–33 years, mean age: 26 years) participated in the angle sorting experiment (the recruitment of participants for this study began on December 1, 2020, and ended on March 5, 2021). All participants were undergraduate students or master’s students from the university community. Handedness was confirmed using the Edinburgh Handedness Inventory. All participants reported normal or corrected-to-normal vision, no history of neurological or psychiatric disorders, and normal tactile sensation as per self-report. No participants had prior experience with haptic psychophysical sorting tasks.

#### Experiment.

The experiment utilized a custom-built haptic angle sorting device described in our previous work [9]. The device featured seven grooves for placing angle stimuli. Stimuli were 2-D raised-line angles made of plastic, with a line width of 1 mm and a height of 0.5 mm. The angles used were 20°, 22°, 24°, 26°, 28°, 30°, and 32° (spanning 22° to 30° in 2° increments). We selected a 2° difference based on pilot studies suggesting it is above the threshold for healthy young adults, thus avoiding floor effects [9,34]. For the sorting task, subsets of these angles were used to create three conditions: 3-angle (22°, 24°, 26°), 4-angle (22°, 24°, 26°, 28°), and 5-angle (22°, 24°, 26°, 28°, 30°) sets. In every trial, the smallest and largest angles of the chosen set were fixed at the leftmost and rightmost positions, respectively, serving as constant spatial anchors to provide a stable reference frame and reduce working memory load.

Participants were seated comfortably in front of the device in a quiet room. To isolate the haptic modality, they were blindfolded throughout the experiment to eliminate visual cues. The heights of the chair and table were adjusted to allow free and comfortable arm movement. At the beginning of each trial, the experimenter placed the fixed anchor angles at the two ends (e.g.,20° and 32° of the 5-angle condition). The remaining angle stimuli were presented to the participant one by one in a pseudorandom order. Ten different presentation orders were pre-generated for each condition (3, 4, and 5 angles). The 30 total trials (10 trials per condition) were administered in a pseudorandomized block design, where the condition for each trial was randomized with the constraint that no more than two consecutive trials were from the same condition.

On receiving a stimulus, the participant actively explored it with the index finger of the dominant right hand. They then used their left hand to place the stimulus into one of the empty grooves between the anchors, aiming to create a left-to-right sequence from the smallest to the largest angle. Participants were allowed to re-explore any already-placed angle and adjust the position of the non-fixed angles an unlimited number of times. Once satisfied with the order, they confirmed the trial, and the sequence was recorded by the device. The experiment followed a within-subjects design with one independent variable: the Number of Angles (3, 4, or 5). The primary dependent variable was the estimated DI derived from each sorting sequence using the MLE model. The time taken to complete each trial was also recorded. Participants performed a total of 30 trials. They were given a mandatory break of at least 5 minutes after every 5 trials to prevent fatigue and maintain concentration. The entire experimental session, including instructions, practice, and breaks, lasted approximately 90 minutes per participant [9,34].

In the experiment where the experimental angles were sorted, compared to the simulation experiments, we only selected 3, 4, and 5 angular stimuli for the sorting task. There are two main reasons for this. Firstly, when using fewer than 3 angle stimuli, the method would require more trials to acquire sufficient data (as in 2AFC). Second, when more than 5 stimuli are used, the decision-making time per trial increases significantly, reducing test efficiency [14,34,37,38]. Based on our repeated experimental measurements, participants were able to judge and confirm the sorting result in less than 3 minutes per trial under these three conditions.

### Data processing and analysis

After each participant completed a sorting trial and confirmed the angle order; the obtained order was recorded for subsequent analysis. Participant details, including name, gender, age, and other characteristics, along with the presentation order of the blocks within each sorting category, were carefully documented. To calculate the DI for each sorting result, we employed the MLE method using MATLAB (MathWorks Inc.). The data were then subjected to statistical analysis using MATLAB (MathWorks Inc.). We used the assessment model described in the paper to calculate the DI for each trial and then computed the average DI after each completed sorting trial. For example, if the DI for the first sorting is $DI_{\text{1}}$ and the DI for the second sorting is $DI_{\text{2}}$, the average DI after the second sorting $DI_{2m}$,is calculated by formula (15), as follows:


$$DI_{2m}=\frac{DI_{\text{1}}+DI_{\text{2}}}{\text{2}}$$ (15)

For the nth sorting trial, the nth average DI,$DI_{nm}$, is calculated by formula(16), as follows:


$$DI_{nm}=\frac{\overset{i=n}{\underset{i=1}{\sum }}DI_{i}}{n},n>1,$$ (16)

where $DI_{i}$ represents the estimated DI of the ith sorting result. The average DIof each participant was also calculated after each trial. We examined the relationship between the stability of the results and the number of experimental trials by calculating the average estimated DI after each trial to determine the optimal number of trials for robust sorting performance. At the same time, we recorded the time each participant spent on completing each sorting task and calculated the average time they took to complete ten trials in order to verify the efficiency of the paradigm in practical applications.

### Results

Compared with the simulation evaluation experiment, this model cannot know the true discrimination performance index DI of the participants in advance of the actual evaluation. Therefore, we attempt to further optimize the experimental parameters based on the stability of the evaluation results through multiple repeated tests of the subjects. In the analysis of the experimental results, we mainly examined the influence of the number of tests and the number of stimuli on the stability of the MLE evaluation model.

**Fig 6:**
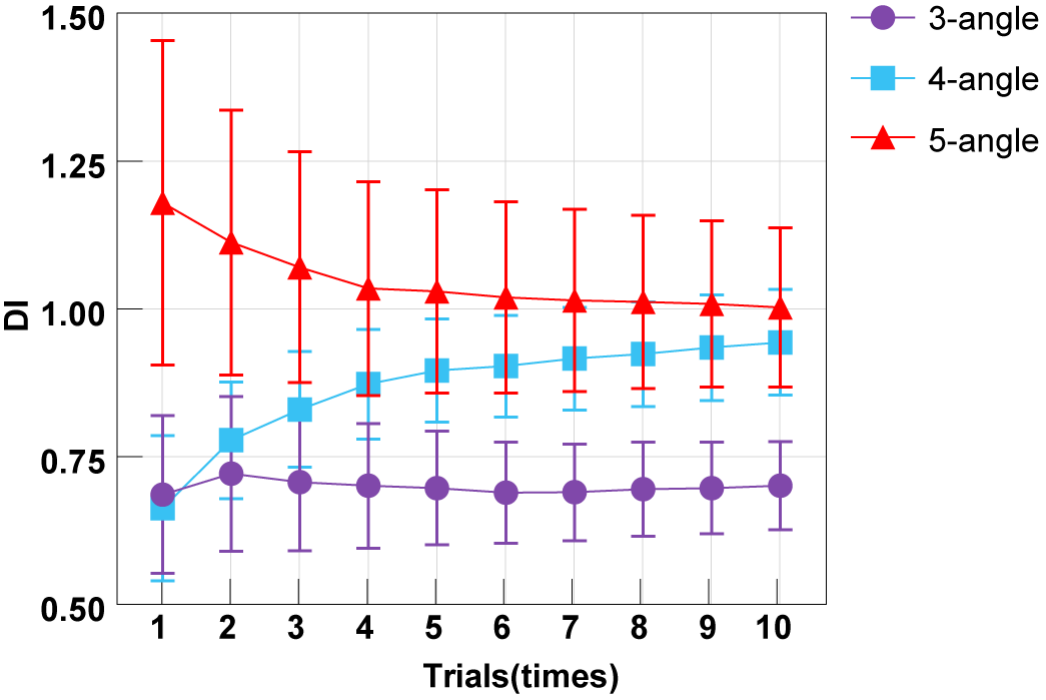
The averageDI calculated by the evaluation model after completing different experimental tasks at 3-5 different angles for all participants.

To study the stability of the number of trials and the number of stimuli on the evaluation results, we calculated the average DI of the estimated values of each trial for all subjects to complete the ranking task with different numbers of stimuli, as shown in Fig 6. The average DI varies with the increase in the number of trials when the number of stimuli is different. When the number of stimuli was 3, the average DI fluctuated slightly between 0.686 and 0.721, without any clear upward or downward trend. The width of the 95% CI significantly narrowed from approximately 0.56 units to approximately 0.32 units; the Cohen’s d values for the changes in DI during the first 10 trials gradually decreased and then increased, ranging from 0.42 (trial 1) to 0.33 (trial 3) and finally to 0.68 (trial 10). The SEMcontinuously decreased from 0.133 to 0.075, a decrease of 43.6%. When the number of stimuli was 4, the average DI monotonically increased from 0.663 to 0.944, with an increase of 42.4%. The 95% CI has shifted upward as a whole (the lower limit has increased from 0.40 to 0.76, and the upper limit has risen from 0.92 to 1.13), and its width has remained relatively stable or slightly narrowed; The Cohen’s d values for the changes in DI during the first 10 trials gradually decreased and then increased, ranging from 0.45 (trial 1) to 0.23 (trial 3) and finally to 0.8 (trial 10). SEMfirst decreased to 0.086 (the 6th time) and then slightly increased to 0.089 (the 10th time). When the number of stimuli was 5, the average DI monotonically decreased from 1.179 to 1.003, a decrease of 14.9%. Meanwhile, the width of the 95% CI has significantly narrowed (from approximately 1.16 units to approximately 0.57 units), and the entire interval has shifted downward and converged; The Cohen’s d values for the changes in DI during the first 10 trials gradually decreased and then increased, ranging from 0.24 (trial 1) to 0.09 (trial 4) and finally to 0.41 (trial 10). The SEMcontinuously decreased from 0.275 to 0.134, a decrease of 51.3%.

**Fig 7:**
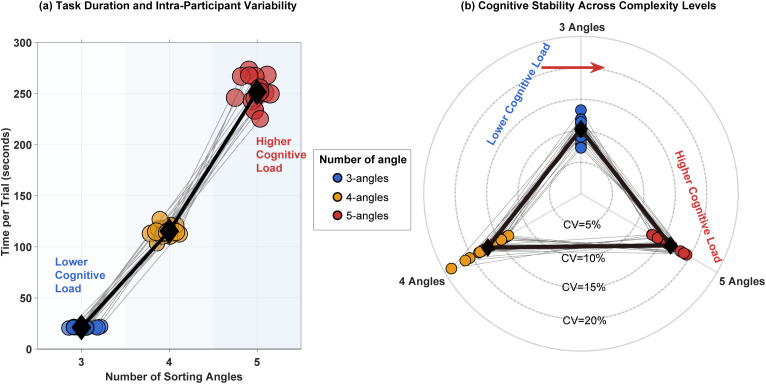
Mean Time per Trial and Number of Sorting Angles Under Different Cognitive Loads. The thin gray lines represent participants; the thick black lines represent Group Mean.

The temporal cost of angular sorting tasks exhibited a near-exponential increase with stimulus complexity, as shown in Fig 7(a). The mean trial duration escalated from 21.3 ± 0.5 s for 3-angle sorting to 115.1 ± 5.3 s for 4-angle tasks, culminating in 252.2 ± 14.9 s for 5-angle configurations (F(2,34) = 824.6, p < 0.001, η² = 0.98). This represents a 12-fold time spent on task when comparing the 5-angle to the 3-angle sorting tasks.

### Discussion

Our data underscore the critical role of repeated trials in stabilizing the DI estimate. Across all stimulus set sizes, the standard error of the DI consistently decreased with more trials, as shown in Fig. 6, aligning with the basic psychometric principle that averaging reduces measurement noise [39–45]. Notably, the most substantial gain in precision occurred within the first three trials, suggesting a practical ‘sweet spot’ for balancing reliability and testing time.

The number of stimuli also affects the deviation of the assessment results and random noise. When the number of stimuli was 3, DItended to be stable (Δ ≤ 2.2%), and the decrease in SEMwas significant (43.6%), but the information volume of DIwas low. Due to the low cognitive load caused by a smaller number of stimuli, random errors dominate the variation. When the number of stimuli was 4, DIincreased significantly (↑42.4%), and SEM rebounded later (>6 times). To a certain extent, the increase in the number of stimuli is dominated by the learning effect, with DIbeing falsely high and its stability decreasing. When the number of stimuli was 5, DItended to decline (↓14.9%), and SEMcontinued to decline (51.3%). Due to the low cognitive load caused by the number of stimuli, random errors dominate the variation. Increasing the number of stimuli enables the high-complexity correction of the initial overfitting and converges towards the true DI.

In addition, the temporal demand of angular sorting tasks exhibited a nonlinear surge with increasing stimulus complexity (Fig 1A). While 3-angle trials averaged 21.3 ± 0.5 s, 5-angle trials required 252.2 ± 14.9 s—an 11.8-fold increase (F (2,34) = 824.6, p < 0.001). The 4 to 5 angle transition alone incurred a 137.1 s penalty (+119%), establishing a critical efficiency bottleneck for real-world applications where testing duration directly impacts feasibility.

## General discussion

In this study, we proposed and validated a maximum likelihood estimation (MLE) framework for assessing perceptual discrimination ability using a sorting task paradigm. The core of this method is the derivation of a DI, which serves as a stable estimator of an individual’s perceptual noise parameter. Through computer simulations and a behavioral experiment on tactile angle discrimination, we systematically investigated how the number of stimuli and trials affects the stability and efficiency of the assessment. The results indicate that the MLE model can reliably recover the underlying parameter under its assumptions, and that in practice, a configuration of five stimuli and three trials offers a favorable balance between assessment robustness and time efficiency for the tactile angle task. This paradigm demonstrates a significant advancement in assessment efficiency, achieving reliable estimates in a fraction of the time required by classic 2AFC methods, as confirmed in our prior comparative study, where the DI from sorting strongly correlated (*r* = 0.9075) with traditional discrimination thresholds [9].

The choice of a sorting paradigm represents a strategic compromise and expansion within the psychophysical toolkit. Traditional forced-choice methods (e.g., 2AFC) are the foundation of psychophysics, providing precise threshold estimates but at the expense of lengthy testing durations due to the requirement for numerous binary trials [14,34–36,46,47]. Adaptive staircases significantly enhance the speed of threshold convergence but typically focus on a single performance point (e.g., 79% correct) and may yield less precise estimates of the underlying psychometric function slope, which is related to perceptual standard deviation (σ) [48–50]. Our sorting paradigm occupies a distinct niche: it is less efficient than a staircase for finding a single threshold but more efficient than 2AFC for estimating the perceptual DI across a defined stimulus range. It does so by capitalizing on the rich, interdependent information contained within a single multi-stimulus arrangement. This makes it particularly suitable for applications where the goal is not only to determine “if” a deficit exists, but to efficiently quantify the magnitude of perceptual noise as a continuous variable, which may be more sensitive to gradual change. Furthermore, the act of sorting multiple items has higher ecological validity for everyday cognitive functioning, as it naturally engages processes like comparative judgment, serial ordering, and working memory maintenance. Consequently, the DI may reflect a composite of perceptual acuity and specific cognitive operations, which can be an asset in screening for conditions where cognitive and perceptual decline are intertwined, such as in neurodegenerative diseases. The paradigm’s simplicity and speed also enhance its practical utility for large-scale or clinical screening, where time constraints and ease of administration are paramount. In the above two experiments, the standard deviation σ of the MLE was employed as an indicator to assess discrimination performance, making the stability of individual experimental outcomes a critical consideration. The two aforementioned experiments primarily focused on optimizing two key parameters—the number of stimuli and the number of sorting trials—to enhance the reliability of individual assessment results. Analysis of these experiments reveals that the stability of the ranked MLE evaluation model is influenced by multiple factors. In the simulation study, several empirically informed constraints were implemented, and correction coefficients were introduced to mitigate model simplification errors. This enabled a comprehensive investigation into the factors affecting both inter-individual and group-level model stability and accuracy, as well as the identification of optimal core parameter settings. Subsequently, in the angle sorting test experiment, guided by the findings from the simulation, the focus shifted to examining the stability of evaluation outcomes for individual participants, with the aim of minimizing significant distortions in evaluation metrics caused by individual variability.

### The number of sorting angle stimuli

The number of stimuli in a sorting task directly influences its informational yield, cognitive demand, and temporal efficiency. Our analysis suggests that five stimuli represent a “sweet spot” balancing these factors.

Firstly, Simulation data indicated that as the number of stimuli increases from three to five, the diversity of possible valid sorting outcomes increases dramatically (from 3 to 63 categories), as shown in Table 1. This greater variety of potential outcomes enhances the model’s discriminative power and reduces the occurrence of invalid (Type III) results, leading to more robust and continuous estimation of the DI. However, the marginal gain in model performance begins to diminish with further increases. Secondly, the behavioral experiment provided critical real-world constraints. As shown in Fig 7, the mean trial completion time increased near-exponentially with the number of stimuli. While a 3-angle task took about 21 seconds, a 5-angle task required approximately 252 seconds—a 12-fold increase. This sharp rise in time is indicative of increased cognitive load, likely involving greater demands on tactile working memory to maintain and compare multiple angle representations. Beyond five stimuli, the escalating time cost and potential for working memory overload would severely compromise the paradigm’s efficiency and practicality for rapid screening or repeated testing. Last but not Last but not least, the experimental results for the 5-angle condition showed that the standard error of the group mean DI decreased consistently across trials, and the mean DI itself exhibited a stabilizing trend. This suggests that five stimuli provide enough data points per trial to allow for stable evaluation value without overwhelming the perceptual-cognitive system.

Therefore, five stimuli strike an optimal compromise: they provide a sufficiently rich dataset for reliable MLE estimation while keeping single-trial duration and cognitive load within acceptable limits for efficient, large-scale, or clinical application [51].

### The number of task trials

The number of repeated trials required is determined by the need to achieve a stable estimate of an individual’s DI while minimizing total testing time.

The simulation study offers clear guidance, as shown in Fig 7(a). The percentage error of DI decreased sharply with the first few trials. Critically, the marginal improvement in estimation precision diminished significantly after approximately three trials. The changes in Cohen’s d from the statistical analysis also further confirmed this result. The reduction in error from the third to the tenth trial was minimal compared to the gain from the first to the third. In addition, in a scenario prioritizing rapid assessment – such as initial screening in a clinical or large-population study-conducting many trials to chase diminishing returns in accuracy is counterproductive. Three trials allow for averaging out random fluctuations in attention or momentary lapses without unduly prolonging the test session. Our behavioral data confirmed that three trials were sufficient to observe a marked decrease in within-condition variability (standard error) for individual participants.

Thus, we propose three trials as a pragmatic standard that captures a substantial portion of the reliability gain offered by repetition, establishing an effective balance between the speed of evaluation and the stability of the resulting DI estimate.

### Efficiency-accuracy-stability tradeoff in angle sorting tasks

In the angle sorting task experiment, the task time consumption increased significantly with the increase in the number of stimulus angles, and at the same time, the cognitive load also increased accordingly. It is worth noting that the increase in task complexity (such as from 3 perspectives to 5 perspectives) has led to a significant increase in the difference in task time consumption among individuals (the standard deviation SD has increased from 2.1 seconds to 41.5 seconds). As shown in Fig 6 (b), 78% of the participants had a coefficient of variation (CV) exceeding the stability threshold of 15% when handling tasks from five angles. This decline in stability can be attributed to working memory overload; the cognitive need to maintain more than five angular relationships has approached the typical limit of working memory capacity [52–56]. An increasing number of angular stimuli can result in two primary effects. On one hand, the variability in individual response times increases (reflected by a higher standard deviation), which undermines the reliability of the test. On the other hand, the extended duration of each trial leads to a diminishing marginal gain in overall testing efficiency. Therefore, this study explores the trade-off between test efficiency and theoretical accuracy based on two experimental findings and identifies the optimal parameters for the haptic angle sorting evaluation paradigm. Furthermore, experimental results indicate that in time-sensitive contexts—particularly when assessing perceptual universality across a broad population—the marginal benefits of improving accuracy by increasing the number of angular stimuli may not outweigh the corresponding efficiency losses.

### Generalizability to other perceptual domains

The MLE – based sorting framework is designed to be modality-flexible in principle, although this requires empirical validation. Its core component is a set of physically ordered stimuli and a task requiring their perceptual arrangement. The principle of optimizing the number of stimuli and trials based on the efficiency-accuracy-stability trade-off is universally applicable. To adapt this paradigm to other domains (e.g., visual line orientation, auditory pitch, or olfactory intensity), two key considerations must be addressed: stimulus set design and parameter calibration. A series of stimuli must be constructed with equally spaced increments along the target perceptual dimension (e.g., orientation in degrees, frequency in Hz). The absolute spacing should be informed by the approximate Just Noticeable Difference (JND) in that domain to ensure the task is neither trivially easy (resulting in prevalent Type II outcomes) nor impossibly difficult (resulting in Type III outcomes). And, the optimal number of stimuli and trials may require slight recalibration. Domains with finer perceptual resolution or lower cognitive load per comparison might efficiently utilize more stimuli. Conversely, domains that are more cognitively taxing might benefit from fewer stimuli. A small pilot study following our simulation-experiment workflow can quickly identify suitable parameters for any new modality or stimulus attribute.

In conclusion, based on the empirical optimization process, we suggest that, at least within the tactile discrimination domain, a configuration of five stimuli and three trials provides a practical balance between efficiency and stability. It captures the essence of the sorting paradigm – a rapid, yet psychometrically sound method for assessing perceptual discriminability. At the same time, this framework provides a possible blueprint for translating the paradigm across sensory modalities, suggesting its potential as a versatile tool for comparative psychophysical research and efficient cognitive screening.

## Limitations and future directions

While the proposed MLE-based sorting paradigm demonstrates promising efficiency and stability in assessing tactile angle discriminability, several limitations must be acknowledged to contextualize its findings and guide its application.

The present validation of the model is confined to a single perceptual attribute (angle) within the tactile domain. The choice of raised-line angles, while well-established in haptics research, represents only one type of spatial cue. It remains an open question whether the model’s performance and the optimal parameters (e.g., five stimuli, three trials) generalize to other tactile features (e.g., curvature, texture, hardness) or to other sensory modalities such as vision (e.g., line orientation sorting) or audition (e.g., pitch sorting). The perceptual sensitivity characteristics and cognitive strategies may differ across modalities and feature types, potentially requiring adjustments to the model or the testing protocol.

The DI is interpreted as a measure of perceptual discriminability. However, the sorting task is a complex cognitive operation that inherently involves more than sensory resolution. It places significant demands on working memory (to maintain and compare multiple tactile representations), attention (to serially explore and integrate information), and executive function (to execute and monitor the sorting strategy). Therefore, the DI likely reflects a composite of perceptual sensitivity and supra-modal cognitive capacity. The observed increase in DI with the number of stimuli (from 3 to 5 angles) may partially stem from increased cognitive load rather than a change in pure perceptual sensitivity. This conflation limits the interpretation of DI as a “pure” sensory threshold and suggests it may be more accurately described as a task-performance index for haptic sorting.

The MLE model derivation implicitly assumes a specific decision strategy – a sequential, pairwise comparison process starting from one end of the continuum. In practice, participants may employ heterogeneous strategies, such as initially identifying the extremes or using a binary insertion method. These alternative strategies could produce different patterns of errors that the current model does not account for. Furthermore, the model assumes that perceptual sensitivity is constant across trials and stimuli. Factors like perceptual adaptation or local changes in exploration force could violate this assumption, introducing bias not captured by the single σ parameter.

Our experiments identified a sharp, non-linear increase in trial completion time when progressing from sorting 4–5 angles. This highlights a practical bottleneck for scalability. While increasing the number of stimuli theoretically enhances the statistical robustness of the DI estimate (as seen in simulations), it comes at a steep cost in testing efficiency and risks participant fatigue in real-world settings. Consequently, the paradigm in its current form is best suited for assessing discriminability using moderately sized stimulus sets where the cognitive load remains manageable. Applying it to very fine discriminations requiring many stimuli may negate its efficiency advantage over traditional methods.

To address these limitations and advance the proposed approach, future research should pursue four directions: cross-modal and multi-attribute validation, disentangling perceptual and cognitive contributions, model refinement and strategy analysis, and neural correlates and clinical application.

A critical next step is to empirically test the model’s validity in other sensory domains and optimize its parameters in sensory domains (e.g., visual grating orientation, auditory frequency) and for other tactile attributes (e.g., spatial frequency, texture). Success across modalities would strongly support the generality of the sorting-based MLE framework for perceptual assessment. Concurrently, developing standardized stimulus sets for these domains would be a valuable contribution.

Moreover, future studies should aim to decompose the standard deviation (σ) in DI scores into perceptual and cognitive components. This could be achieved by employing dual-task paradigms (e.g., performing a sorting task while holding a verbal memory load) to explicitly manipulate cognitive demand. And correlating DI scores with independent, standardized measures of working memory capacity and executive function. Comparing DI from the active sorting task with thresholds obtained from a passive, simplified discrimination task (e.g., a classic 2AFC) that minimizes strategic and memory demands, to isolate the “core” perceptual standard deviation (σ).

In addition, the computational model can be enhanced by incorporating process-tracking data, such as hand movement kinematics or eye-tracking in visual tasks, to infer individual sorting strategies. This data could be used to validate the model’s assumptions or to develop more sophisticated, strategy-aware models. And exploring alternative underlying noise distributions (e.g., Logistic) within the MLE framework and comparing their fit to empirical data. And developing adaptive versions of the sorting paradigm where the stimulus spacing is dynamically adjusted based on ongoing DI estimates, minimizing the occurrence of uninformative trials (akin to Type III results) and further improving efficiency.

Investigating the neural underpinnings of the sorting task using neuroimaging (fMRI, EEG) can solidify its theoretical foundation. Key questions include: Which brain networks (somatosensory, prefrontal, parietal) are most predictive of DI? How does neural activity change with increased cognitive load during sorting? Furthermore, the clinical utility hinted at in prior work must be rigorously tested. Longitudinal studies involving populations with Mild Cognitive Impairment (MCI), Alzheimer’s disease, or peripheral neuropathies are needed to establish the sensitivity, specificity, and predictive value of the sorting-based DI for early detection and disease progression monitoring.

By systematically addressing these limitations and pursuing these future directions, the MLE-based sorting paradigm can evolve from a promising proof-of-concept into a robust, versatile, and clinically valuable tool for the efficient assessment of perceptual and cognitive function.
